# Analysis of the Elnady Technique on Cadaveric Tissues: A Low Barrier-to-Entry to Increase Tissue Handling Comfort, Longevity, and Safety

**DOI:** 10.7759/cureus.99483

**Published:** 2025-12-17

**Authors:** Grant G Harris, Jeremy M Jones, Thomas G McNary

**Affiliations:** 1 Anatomy Department, Alabama College of Osteopathic Medicine, Dothan, USA

**Keywords:** elnady technique, medical education, plastination alternative, prosection preservation, tissue shrinkage

## Abstract

The Elnady technique, glycerin impregnation, stands to ameliorate a cost problem by allowing the preservation of prosection specimens in-house. When using this technique, some shrinkage is observed after dehydrating the tissues with acetone. To quantify this and to demonstrate the technique, three human brains (one whole brain, one hemisection, and one hippocampus dissection) and one heart were dehydrated using >99.5% acetone baths at room temperature; baths were changed weekly with hydrometer readings to measure purity. Organs were then immersed in glycerin baths for two weeks, removed, drained, and placed in cornstarch for curing. Dimensional measurements of organs were taken before and after treatment. There was shrinkage of the heart and brain circumference by approximately 10%, with similar shrinkages in other dimensions. Acetone removed much of the fat left after dissection of the heart and caused significant hardening of the aorta, which softened after exposure to glycerin and subsequent curing with cornstarch. Twenty-four months later, a set of three whole brains were impregnated with glycerin using the same methods. The mass, volume, and circumference were measured before and after treatment. The whole brains were dissected to expose the hippocampus. No acetone was found in the lateral ventricles of the whole brains. The whole brain impregnated with glycerin 24 months prior no longer smelled of acetone, while the recently treated brains did. At least six days after treatment, the mass of the recently treated whole brains had decreased.

## Introduction

Though the need for donor specimens in medical education is constant throughout the world, access to human donors can be a challenge [1]. Additionally, fixation during the initial embalming process can be inconsistent. Thus, specimens retained for extended timeframes after prosection for instruction and examination purposes are often preserved via formalin/formaldehyde-based solutions. These solutions often irritate the eyes and upper airways and pose health risks to those who frequently handle them, including specific cancers [2,3]. Plastination has been around for several decades but requires a degree of expense. For example, one must purchase or manufacture dedicated equipment and space [4]. Nonbiologic alternatives such as models or 3D virtual apps can help to teach basic structure recognition and relationships, but medical schools have sustained anatomy departments, reflecting that quality and quantity of real laboratory dissection is necessary [5]. At the end of an anatomy course, many donors’ cremains are sent to family or friends, in accordance with the consent of the living donor. However, some donors consent to permanent donation, e.g., without any time constraint. The Elnady technique can help improve long-term preservation of prosection specimens in-house, while requiring few resources and equipment, and leaving tissues dry, malleable, and free of chemical irritation for handlers and learners [6]. This allows an anatomy program to be a better steward of a donor’s original intent in leaving a permanent donation.

Although the Elnady technique was first developed for use in veterinary schools [6-8], medical schools utilize donor specimens to teach gross anatomy with similar protocols, which are first embalmed. To do this, a fixative (e.g., formalin, formaldehyde, xylene) is introduced into a major artery using the pressure created by an embalming pump or gravity; an associated large vein is also cannulated to remove returning material from vasculature [9]. This fixation is typically suitable to preserve human donor tissue for dissection, yet dissecting embalmed bodies releases embalming compounds. Through physical contact and inhalation, a strong volatile smell can irritate the eyes and upper airways [2].

Formalin, formaldehyde, or Carosafe® (Carolina Biological Supply Company, Burlington, NC, USA) are typically used when preserving a prosection for future courses. Although the former two chemicals are very common, they are known carcinogens, posing health hazards to faculty and students [2,10]. Some schools utilize Carosafe® baths after tissue is removed from the donor's body. Many schools use models as a replacement or supplement to teach basic structure recognition and relationships, which solves the chemical hazard problem but introduces other limitations like errors, idealistic representation of non-ideal structures, and unrealistic firmness. Plastination specimens overcome the first two limitations and have many of the benefits of prosections, which can compete with dissection as a learning aid [11]. 

The traditional von Hagens plastination process consists of fixation using embalming or a similar process, dehydration and defatting using acetone or a similar volatile liquid, forced impregnation using proprietary plastics or silicones, and finally curing [12]. Depending on the scale of plastination, the flammable and combustible liquids used to treat tissue can create an explosion risk; mitigating these risks may require large monetary investments in spaces specifically designed to accommodate the process [13,14]. Though there are still risks to using volatile, flammable chemicals such as acetone, these are mitigated through smaller scale of fixation (i.e., fixing a select organ in contrast to a whole body), proper handling, proper ventilation, and the use of sealed containers. 

The Elnady method seeks to strike a balance between preservation of specimens for future students and supporting the dissection-based anatomy program. Glycerin impregnation allows small anatomy programs a cheap and safe way to preserve human donor specimens on a small scale with easily accessible materials and conditions, in only a typical, room-temperature laboratory environment; it yields “durable and flexible” specimens without expensive and often custom-made equipment [6,7,15]. The required agents (acetone as a dehydration agent, glycerin as a preservative, and cornstarch to draw out the excess glycerin) are all commonly available and relatively inexpensive and provide an alternative to both wet preservation and use of anatomic models while utilizing permanently donated tissues [15].

Reihl et al. [15] gathered morphometric data on central neuroanatomical structures as well as the superficial temporal artery. Of the brain preparations they used, a whole brain preparation demonstrated latex injection to provide contrast and coloring to Elnady specimens; they also demonstrated effects of the Elnady method on brain specimens peeled using the Klingler technique [15,16]. This paper aims to further elaborate on the change of mass specimens experience as acetone evaporates after the application of the Elnady technique. We applied the Elnady technique on similar brain preparations (a whole brain, a hemisected brain, and a hippocampal dissection), as well as preparation of the heart. This is the first report of using the Elnady technique on a human heart. Hopefully, these preparations add to the useful measurements for predicting the differential shrinkage in brain and myocardial tissues. 

## Materials and methods

We used human donor specimens that were previously fixed using the embalming protocol of the Alabama College of Osteopathic Medicine willed body program. These specimens were obtained through ethical means, each dissected from a permanent donor, meaning someone who gave their living consent to be studied without any time constraint. Specimens underwent dissection in accordance with the Anatomical Sciences course at this institution. 

All specimens were originally fixed with the proprietary embalming protocol and were transferred to Carosafe® months prior to applying the Elnady technique as originally described. Transfer of brains from formalin solutions to Carosafe® was originally completed by lab staff without anticipation of them being included in this research.

Dehydration of each preparation began with a one-week bath of >99.5% acetone, ensuring that the organs were completely immersed within a closed container at room temperature. After a week, the concentration of acetone was then measured, and the bath was renewed. Once the concentration remained at 98-99%, the specimen was deemed sufficiently dehydrated. Before glycerin impregnation, the organs were dabbed dry of acetone and allowed to drain on a sieve. Using a similar closed container, they were immersed in glycerin for four weeks for certainty. Although the original Elnady paper only described a maximum of two weeks depending on composition [7], there is a precedent for leaving organs in glycerin for longer periods of time [6]. The specimens were immersed in glycerin by putting each specimen in a mesh bag, which contained additional weights to ensure adequate submersion.

The organs were then drained of the glycerin for two days, placed in a cheesecloth bag, and covered with ground cornstarch to draw out the excess glycerin. When cornstarch came in contact with glycerin, it clumped. The clumped cornstarch was removed, and the bags were turned intermittently until the cornstarch stopped clumping. Before presenting, the organs were brushed with a soft brush and gently dusted with compressed air.

Preparations

Dissection of one human heart with associated aortic arch was completed to remove the pericardium and connective tissue. The pericardial fat surrounding the heart was mostly left intact. Chambers were opened and blood was removed. 

In the first phase of the experiment, dissection of three human brains was completed post-embalming. One brain was left whole, overlying arachnoid mater were left intact except for damage sustained by the bone saw during removal. One brain was hemisected, including the brainstem and cerebellum. The third brain underwent dissection by faculty to highlight the lateral ventricle, hippocampus, and other associated structures. The pieces that were removed to expose the hippocampus were kept with the dissected brain while applying the Elnady technique.

All specimens were measured using a measuring tape or dial calipers during the first phase. Specimens were measured pre-treatment and post-treatment. The same instruments were used for each organ before and after the Elnady technique. Calipers used were new Pittsburgh 6-inch dial calipers converted from standard to metric units. Circumference was measured using the same disposable tape measure before and after, so as to preserve reliability of the shrinkage percentage. Both circumference and length were measured at their respective highest values (e.g., from occipital pole to frontal pole for the brain, circumference of the ventricles for the heart).

In the second phase of the experiment (24 months later), three whole brains were measured and treated as the first whole brain specimen with the following exceptions. The mass of the whole brain specimens was measured before dehydrating with acetone, impregnating with glycerin, after treatment with cornstarch, and again at least six days after treatment was completed. A hippocampal dissection was then performed. The volume was measured using water displacement before dehydration in acetone and again before placing the specimens in cornstarch.

## Results

Heart

**Figure 1:**
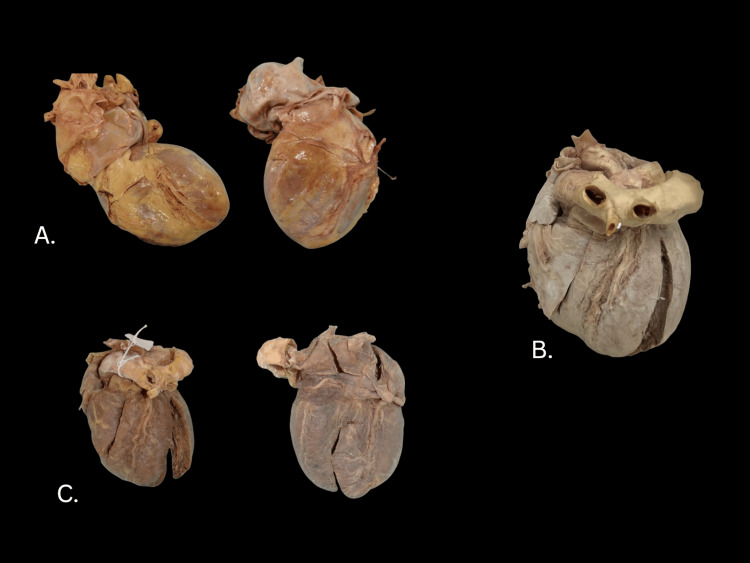
Images of the donor heart preparation during various stages. A: Pre-treatment, donor heart, pericardium reflected upward. B: Post-acetone treatment. C: Completed Elnady treatment.

Before treatment, the heart specimen had abundant pericardial fat (Figure 1A). The color of the myocardium blanched considerably after dehydration (Figure 1B) but regained its darker color after glycerin impregnation (Figure 1C). At the end of processing, there was no residual acetone smell. The attached aortic arch hardened considerably during dehydration but regained most of the original flexibility following exposure to glycerine and curing with cornstarch. Acetone dehydration at room temperature also removed most of the associated visceral fat from the organ, clearly exposing the coronary arteries. The right ventricle reduced in thickness by 5.7% compared to the left ventricle at 9.1% (Table 1).

**Table 1:** Changes in heart dimensions, comparing measurements before and after applying the Elnady treatment. RV: right ventricle, LV: left ventricle

	Before Elnady	After Elnady	Difference	% Change
Base Circumference	29.0 cm	25.5 cm	-3.5 cm	12.1 %
Length	12.5 cm	11 cm	-1.5 cm	-12.0 %
Diameter of Aorta	2.0 cm	2.0 cm	0.0 cm	0.0 %
RV Wall Thickness	0.35 cm	0.33 cm	-0.02 cm	-5.7 %
LV Wall Thickness	0.66 cm	0.60 cm	-0.06 cm	-9.1 %

Whole brain

**Figure 2:**
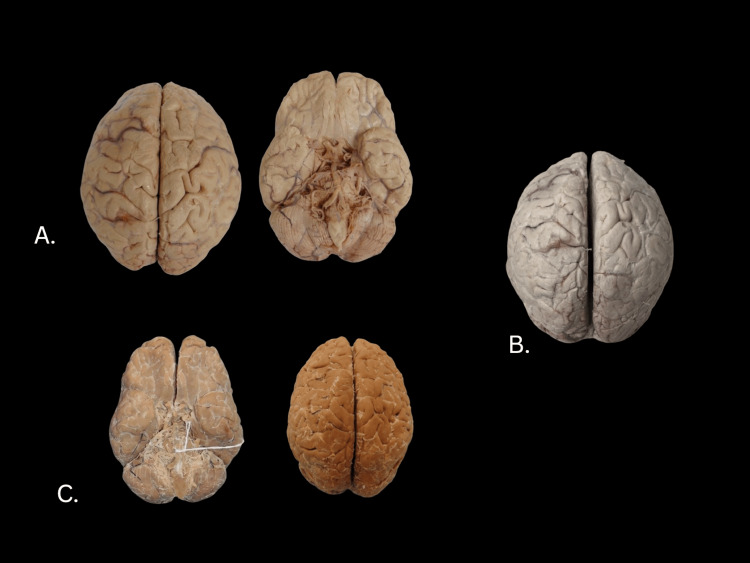
Images of the donor whole brain preparation during various stages. A: Pre-treatment, donor whole brain. B: Post-acetone treatment. C: Completed Elnady treatment.

Before treatment, the whole brain specimen had been stored in Carosafe® (Figure 2A). After dehydration, the tissue blanched considerably (Figure 2B). After impregnating the brain with glycerin, the color became considerably darker (Figure 2C), even becoming darker than the specimen prior to dehydration. Within weeks after treatment the tissue was dry and elastic but retained some of the acetone odor that was noticeable when removed from its storage bag. However, the odor was significantly less than what was observed throughout glycerin impregnation. There was no residual formalin or formaldehyde odor.

Hemisected brain

**Figure 3:**
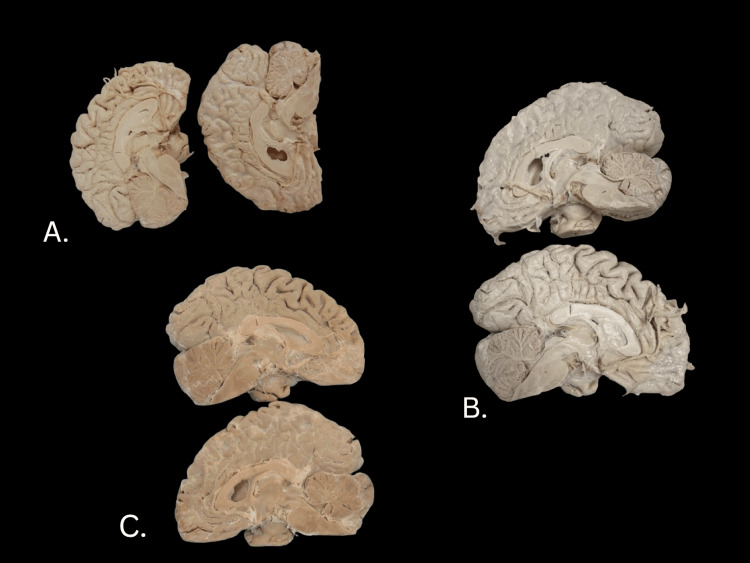
Images of the donor hemisected brain preparation during various stages. A: Pre-treatment, donor brain hemisection. B: Post-acetone treatment. C: Completed Elnady treatment.

The specimen was dry, elastic, and retained no acetone, formalin, or formaldehyde odor once the process was complete. Like the whole brain, the hemisected brain (Figure 3A) blanched considerably during dehydration (Figure 3B) and then darkened following the glycerin impregnation (Figure 3C). The final color was also considerably darker than the original specimen that was stored in formalin.

Hippocampus dissected brain

**Figure 4:**
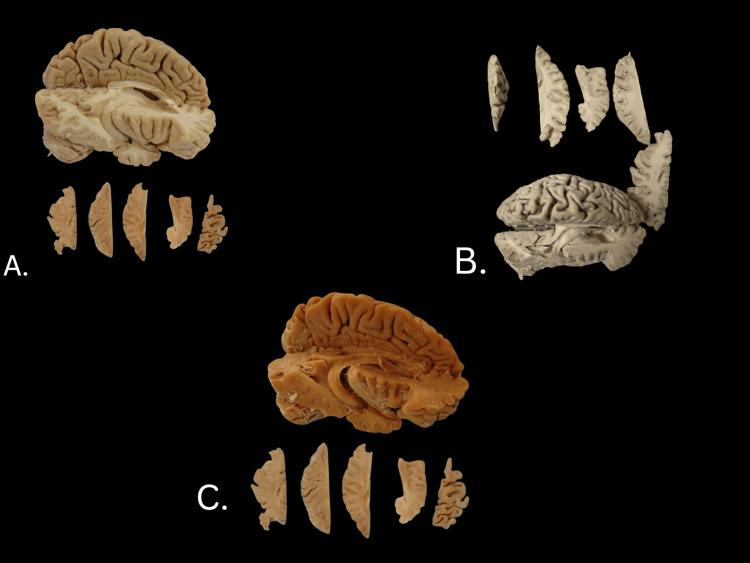
Images of the donor hippocampal brain dissection during various stages. A: Pre-treatment, donor hippocampus. B: Post-acetone treatment. C: Completed Elnady treatment.

The specimen was dry, elastic, and retained no acetone, formalin, or formaldehyde odor once the process was complete (Figure 4). Like the other brain specimens, the hippocampus dissected brain (Figure 4A) blanched considerably during dehydration (Figure 4B), followed by darkening during glycerin impregnation (Figure 4C). The final color was considerably darker than the original specimen. Notably, the cortex shrank surrounding the adjacent ventricle, causing the collapse of the opening to the left lateral ventricle. None of the other brain specimens experienced a collapsed interventricular foramen. The changes in brain dimensions are recorded in Table 2. Generally, the specimen dimensions shrank by about 10%.

**Table 2:** Changes in brain specimen dimensions, comparing measurements before and after applying the Elnady treatment.

	Before Elnady	After Elnady	Difference	% Change
Whole Brain Circumference	49.5cm	44.7cm	-4.8cm	-9.7%
Hemisected Brain AP Length	17.0cm	15.2cm	-1.8cm	-10.6%
Hippocampus AP Length	16.5cm	15.2cm	-1.3cm	-7.9%

Phase two

After removing each whole brain specimen from acetone, prior to placing the brains in glycerine, the mass was unstable, making a reliable report of mass difficult. The range of change between specimens was around 50-60% of the original mass, but kept decreasing. The mass of the whole brain specimens post-glycerine impregnation was close to the original mass (Table 3), then the mass dropped to around 20% post-treatment (after removing the brains from cornstarch). Six days after the conclusion of the treatment the mass further decreased. The tissue retained an acetone scent. By dissecting the hippocampus on one side for each of the whole brains, we determined there was no acetone reservoir in the lateral ventricles.

**Table 3:** Mass of three whole brains, before and after Elnady treatment.

	Mass (before treatment)	Mass (post-glycerin treatment)	Mass (post-treatment)	Mass (six days post-treatment)
Brain #2	1143 g	1066 g	913	871 g
Brain #3	1212 g	1214 g	986	909 g
Brain #4	1158 g	1200 g	937	898 g
Average	1171 g	1160 g	945	893 g
Standard Deviation	36.3 g	81.7 g	37.2	19.6 g
Average Percent Change	-	-5.8%	-19.3%	-23.7%

The average shrinkage of the whole brain specimens, as measured by circumference, was similar to the original whole brain, averaging in the single digits of percent (Table 4). The average change in volume was -8.80%.

**Table 4:** Volume and circumference of three whole brains, at selected points during the Elnady process.

	Volume (before treatment)	Volume (post- glycerin treatment)	Circumference (before treatment)	Circumference (post-treatment)
Brain #2	1205 mL	1073 mL	48.5 cm	44.7 cm
Brain #3	1250 mL	1149 mL	48.2 cm	45.8 cm
Brain #4	1240 mL	1148 mL	47.7 cm	44.5 cm
Average	1232 mL	1123 mL	48.0 cm	45.0 cm
Standard Deviation	23.6 mL	43.6 mL	0.4 cm	0.7 cm
Average Percent Change	-	-8.80%	-	-6.51%

The original whole brain was also dissected to show the hippocampus on one side, and it no longer had any remaining acetone scent.

## Discussion

The initial three brain specimens were dissected differently from each other with the intention of developing specific teaching prosections. The differences between these specimens posed some unexpected outcomes. For example, the hippocampal specimen (Figure 4) only required three acetone baths to meet the hydrometer threshold, whereas the whole brain (Figure 2) required five. Also, the hippocampal specimen had collapsed at the interventricular foramen, while the whole brain specimen retained the scent of acetone, making it unclear if there was a reservoir of acetone within the lateral ventricles. These outcomes during the first phase led to the second phase of experiments.

We determined that treating whole brain specimens would reduce the likelihood of the interventricular foramen collapsing as it had for the hippocampal specimen. Thus, we planned to perform a hippocampal dissection after treating the whole brain specimens in the second phase. Cutting glycerin-impregnated brain tissue for the hippocampal dissection was more challenging than cutting brain tissue only fixed with formalin. The glycerin-impregnated tissue was firmer than formalin-fixed tissue. Yet, the interventricular foramina in each case was patent, making a more favorable prosection for teaching purposes. We also found there was no reservoir of acetone within the lateral ventricles in situ, suggesting the acetone was only interstitial. Each whole brain specimen continued to decrease in mass six days after completing the Elnady technique (Table 3). This loss of mass was more likely due to acetone evaporation than loss of glycerin as no residual glycerine outside the tissue was noticeable on storage containers. After two years, the residual acetone odor of the original whole brain specimen was gone.

Assuming the total water concentration was similar between the hippocampal and whole brain specimens prior to treatment, the difference between these two preparations is likely due to associated kinetics related to distance for diffusion. Therefore, more water was removed per acetone bath for the specimen with higher surface-to-volume ratio. Similarly, the acetone evaporated more slowly from the specimen with the lower surface-to-volume ratio. Analogously, performing a hippocampal dissection on a whole brain, post-glycerin impregnation, should accelerate the evaporation of acetone by reducing the total volume. 

We verified that the changes in dimensions of the whole brain specimens during the second phase were similar to the changes in dimensions of the first phase, two years prior. We did not measure the volume of the whole brain specimens with water displacement post-acetone or post-cornstarch to avoid potentially re-impregnating the tissue with water. There was a decrease in mass that occurred after dehydration in acetone, before placing the specimens in glycerin. Ultimately the changes in measurements indicate that the mass (Table 3), volume (Table 4), and linear dimensions (Table 2) decreased after complete implementation of the Elnady technique. Loss of excess glycerine occurred while drying with cornstarch and will likely continue at a slower rate as the specimens are handled, though not nearly at the rate of the acetone. 

The initial specimens proved useful for learning; the smell, durability and flexibility allow quality prosections to be reused each year. Even the differential shrinkage might help in separating components that need to be identified separately. For example, the exposure of the coronary arteries in the heart prosection was a particular strength. The visceral fat was removed by the acetone (Figure 1), which made coronary artery identification easier for the students. The epicardial fat that was present on the heart specimen prior to dehydrating the heart appeared absent after treatment; this is much like the use of acetone for defatting in typical plastination. 

In general, however, differences from the in vivo organ are not without limitation. For the heart specimen, the difference in shrinkage of the right ventricle versus the left ventricle could be due to differences in tissue alignment, the right ventricle having a longitudinal orientation and the left ventricle having twist mechanics associated with the cardiac apex’s rotation [17]. Further limitations include the relatively small number of organs (which are too small to have a meaningful analysis of variance) and the failure to measure mass during the first phase. Though grossly similar, the changes in volume and circumference are not closely related or predictable based on the initial size of the brain. The selection of brains was done based on availability; no effort was put into controlling for sex, pathology, etc, and there are significant differences in the anatomy of brains between males and females, females having significantly more grey matter, and differences in sheer size of components, such as the corpus callosum, amygdala, etc. [18]. Interestingly, in the brain specimens, there was qualitatively very little difference in the contrast between the gray and the white matter whether the dissection had occurred before or after being fixed in glycerin.

Future studies may consider variations on the Elnady technique. For example, one variation on the Elnady technique may involve immersing the organ in cold acetone (-25°C) before impregnating the tissue with glycerin [19,20]. A benefit of dehydrating with cold acetone is less shrinkage [21]. Alternatively, incrementally higher concentrations of ethanol baths up to 100% may also be used before impregnating the tissue with glycerin. However, this approach is described as more time-consuming, and the amount of tissue shrinkage is greater [22].

The longevity of the Elnady process specimens has been shown to last at least four years, and likely more [15], though the original Elnady publication is still recent. It is difficult to compare the longevity of traditional plastination, Elnady plastination, and anatomical models, as their longevity will have different limitations. Because the cost to glycerate tissue is so much lower than plastination [15], it may be easier to justify the cost to use an organ at least four years longer, which would be in harmony with the body donor’s intentions [23].

## Conclusions

The Elnady technique or glycerine impregnation is an economical option for universities to preserve permanent donations for future learning and consistency in examination, in a flexible form without the harsh formaldehyde mixtures largely used presently. Plastination proper is the gold standard in longevity, though it is expensive and stands to supplant the use of traditional dissection, rather than support it. The Elnady technique allows for even better visualization of coronary arteries in heart preparations compared to traditional dissection alone and is relatively inexpensive. 

This paper sought to: validate the utility of the technique, refine the process itself, and illustrate some of the limitations related to acetone evaporation from tissues with lower surface-to-volume ratio. It is important to consider the donation people give of themselves when deciding to contribute to medical education and research; use of this technique enables a donor’s gift to educate more students, further enabling their wishes. Besides the study of quantitative changes of mass and dimensions and qualitative observations of color and composition, the final goal was to create an anatomical specimen for teaching, which was demonstrated successfully at our institution.
